# Distribution of Cenozoic plant relicts in China explained by drought in dry season

**DOI:** 10.1038/srep14212

**Published:** 2015-09-15

**Authors:** Yongjiang Huang, Frédéric M. B. Jacques, Tao Su, David K. Ferguson, Hui Tang, Wenyun Chen, Zhekun Zhou

**Affiliations:** 1 Key Laboratory for Plant Diversity and Biogeography of East Asia, Kunming Institute of Botany, Chinese Academy of Sciences, Kunming 650201, China; 2 Key Laboratory of Tropical Forest Ecology, Xishuangbanna Tropical Botanical Garden, Chinese Academy of Sciences, Mengla 666303, China; 3 Institute of Palaeontology, University of Vienna, Althanstrasse 14, Vienna A-1090, Austria; 4 Department of Geosciences and Geography, University of Helsinki, Helsinki FL 00014, Finland

## Abstract

Cenozoic plant relicts are those groups that were once widespread in the Northern Hemisphere but are now restricted to some small isolated areas as a result of drastic climatic changes. They are good proxies to study how plants respond to climatic changes since their modern climatic requirements are known. Herein we look at the modern distribution of 65 palaeoendemic genera in China and compare it with the Chinese climatic pattern, in order to find a link between the plant distribution and climate. Central China and Taiwan Island are shown to be diversity centres of Cenozoic relict genera, consistent with the fact that these two regions have a shorter dry season with comparatively humid autumn and spring in China. Species distribution models indicate that the precipitation parameters are the most important variables to explain the distribution of relict genera. The Cenozoic wide-scale distribution of relict plants in the Northern Hemisphere is therefore considered to be linked to the widespread humid climate at that time, and the subsequent contraction of their distributional ranges was probably caused by the drying trend along with global cooling.

Global climatic changes are causing important concerns about biological changes^1^,^2^. To avoid a future biodiversity collapse, we need to understand the influence of climatic changes on organisms. Palaeontological studies provide an important temporal framework to address these questions, because they can consider former ecology of the nowadays extinct species. However, understanding the causes of biotic extinction is not an easy matter, due to the lack of information about the climatic requirements and physiology of extinct organisms.

Cenozoic plant relicts are those groups that were once widespread in the Northern Hemisphere but are now extinct in most of their former geographic ranges^3^,^4^,^5^ (Fig. 1). They are therefore good models to study how past climatic changes have caused some plants to contract or even become regionally extinct. Among the Cenozoic climatic changes, the Quaternary glaciations are thought to be one of the most profound events influencing modern plant diversity and distribution^4^,^5^,^6^,^7^. This is particularly conspicuous in Europe where the Quaternary glaciations have largely shaped the modern diversity, distribution and genetic structure of plants^8^,^9^,^10^. During the last glacial period, the European distribution of many tree species was restricted to a few refugia, e.g., the Mediterranean regions^8^,^10^,^11^. In contrast to the situation in Europe, in China with relatively low latitudes many Cenozoic relict genera have successfully survived^5^,^12^. They occur mainly in biological hotspots that are considered as refugia for their survival and population^12^,^13^, but some of them are classified as endangered lineages. The present distribution of relict plants in China can also be explained by the role of the Quaternary glaciations^14^. During the Last Glaciation Maximum, the snow line in central China was estimated to be 2500–2600 m^15^,^16^. In southeastern China, glaciers might have been present but restricted to high altitudes in mountainous areas^17^. Even if the Quaternary glaciations can explain the extinction of Cenozoic relict genera in Europe, they cannot really explain the modern distribution patterns of such genera in some regions of China.

Cenozoic plant relicts are also of interest in connection with biodiversity conservation. They were once important elements of the northern hemispheric floras^3^,^4^,^5^. The subsequent contraction of their distributions was not caused by human activities; however, they are now easily threatened as they are restricted to small isolated areas. Studying the modern distributions of Cenozoic plant relicts and the relationship with the climatic conditions of their habitats can be an important step towards their conservation.

In this study, we look at the modern distributions of Cenozoic relict genera in China to recognize their best refugia. We identify the climatic requirements of these Chinese relict genera to explain their modern distribution pattern in China, and then compare the climatic conditions of the modern and past distributions of the Cenozoic relicts to understand the historical contraction of their distributional ranges.

## Results

### Modern climate of China

**Figure 2:**
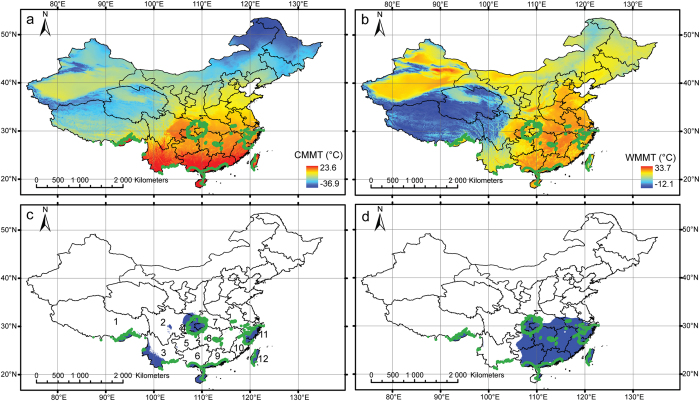
Modern climate of China, with the green line representing regions where both autumn and spring precipitations are over 300 mm: (**a**) cold month mean temperature, (**b**) warm month mean temperature, (**c**) autumn precipitation, regions with autumn precipitation over 300 mm are in blue, (**d**) spring precipitation, regions with spring precipitation over 300 mm are in blue. (1) Tibet, (2) Sichuan, (3) Yunnan, (4) Chongqing, (5) Guizhou, (6) Guangxi, (7) Hubei, (8) Hunan, (9) Guangdong, (10) Fujian, (11) Zhejiang, (12) Taiwan. Maps were generated using the software ArcGIS 9.3.

In winter, there is an obvious latitudinal temperature gradient in China, with lower temperatures in the north and higher temperatures in the south (Fig. 2a). In summer, eastern China is warm to hot, whereas western China is cool, especially in the Qinghai-Tibetan Plateau due to high altitudes (Fig. 2b).

The precipitation pattern is more complex than the temperature pattern. In autumn, only central China and some isolated regions in southern and southeastern China are humid (Fig. 2c). In spring, a wide region from central to southeastern China has a humid climate, with a precipitation value of at least 300 mm (Fig. 2d). Only a few regions are humid in both autumn and spring; they are primarily in central China (Chongqing Municipality, eastern Sichuan and western Hubei provinces), followed by some isolated regions including southeastern Zhejiang province, Taiwan Island, southernmost China, and southern slopes of the Himalayas.

### Distribution and hotspot of Cenozoic relict plants

**Figure 3:**
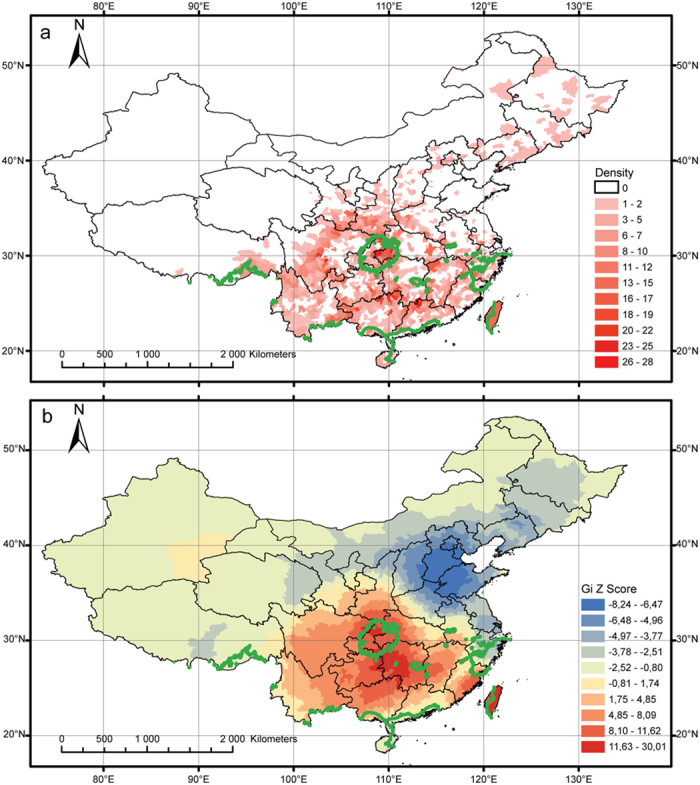
Distribution of relict genera in China, with the green line representing regions where both autumn and spring precipitations are over 300 mm: (**a**) number of relict genera per county, (**b**) results of the hotspot analysis, with red colours indicating hotspot regions and blue colours indicating cold-spot regions. Maps were generated using the software ArcGIS 9.3.

Figure 3 shows the number of Cenozoic relict genera present in each county. The counties with high numbers of relict genera are situated mainly at the border of Chongqing and Hubei, those of Hunan, Guangxi and Guangdong provinces, and the Tibetan margin of Sichuan (Fig. 3a). The county with the highest number of relict genera is Nanchuan in Chongqing (28 genera), followed by Lichuan in Hubei and Ruyuan in Guangdong, both with 26 genera.

Hotspots of Cenozoic relict genera are found primarily at the border of Chongqing and Hubei, the border of Guizhou and Hunan, and eastern Fujian and Taiwan Island (Fig. 3b). These correspond to hotspots that have already been described: the mountainous central China including eastern Chongqing and western Hubei and the mountainous regions of northern Guangxi, southeastern Guizhou and southwestern Hunan^12^, plus Taiwan Island. The central China hotspot corresponds to the centre of palaeoendemic diversity^13^.

Detailed contributions of each climatic parameter to explain the distribution of each relict genus are given in Supplementary Tables S2 and S3. Precipitation parameters are shown to play an important role in controlling the distribution of relict genera (Supplementary Table S4).

## Discussion

In China, the precipitation regime is controlled primarily by the Asian monsoon system^18^. It is characterized by dry winters and wet summers^19^,^20^. In spring, the rain known as Plum rain progressively enters China from the southeastern coast^21^. The rain arrives later in western China, which potentially explains why this region shows lower precipitation in spring (Fig. 2). In winter, cold high pressures over Siberia and Mongolia create cold dry winds over China^22^,^23^. However, the decrease of precipitation observed in autumn is less pronounced in central China and Taiwan Island. These two regions have a shorter dry season than other parts of the country.

As the Cenozoic relict genera in this study belong to different families, a phylogenetic explanation can be excluded. Hence a climate associated factor is more probable to explain the overlapping ranges of these relict genera. The temperatures, in terms of mean annual temperature, winter and summer temperatures, which are now recorded from central China and Taiwan Island are comparable to those observed from many other places of China. In other words, the climate of these regions cannot be discriminated from the climate in other Chinese regions based only on temperatures. Besides, the fossil distributions of some relict genera in China imply that they were more widespread in the past, and could withstand a larger range of temperature. For example, *Metasequoia* was found from the High Arctic to Europe during the Paleogene^24^,^25^, demonstrating its wider temperature-tolerance than that estimated from its modern range. The temperature therefore is unlikely the limiting factor explaining the present distribution of relict genera. The species distribution models also indicate that the temperature-related parameters only explain the distribution of a few relict genera, especially those with a southern distribution. Understanding the modern distribution of relict genera needs to go beyond the temperature parameters.

On the contrary, the precipitation pattern is more closely correlated with the distribution of Cenozoic relict genera. As demonstrated by the species distribution models, the precipitation parameters are the most important explanatory factors for more than half of the relict genera, especially some flagship taxa such as *Cercidiphyllum* and *Metasequoia*. Central China and Taiwan Island have high precipitations in both autumn and spring, corresponding to palaeoendemic hotspots as they are. Having humid conditions, the southern slopes of the Himalayas are characterized by tropical vegetations, similar to Indian vegetations, and thus are beyond the scope of this study. Zhejiang and southernmost China are more urbanized, and natural vegetations are rare, making a credible link between the natural vegetation and climate problematic. To conclude, palaeoendemic hotspots are situated in regions where the dry season is shorter and both autumn and spring have high precipitations. We therefore hypothesize that the length of the dry season is a key factor explaining the distribution of relict genera. Central China and Taiwan Island, having a shorter dry season, can provide suitable refugia for relict genera that are unable to conquer seasonal drought. This may be the first suggested link between humidity and the distribution of Cenozoic relict plants.

Because a large spectrum of genera of both gymnosperms and angiosperms is included, the physiological aspects of the seasonal drought intolerance may be different from one genus to another. Several hypotheses can be made concerning this intolerance. Among the angiospermous and gymnospermous genera studied here, most have their seeds germinate in spring, and some have seeds without dormancy and can germinate immediately after maturity in autumn^26^ (Supplementary Table S1). In central China and Taiwan Island with humid autumn and spring, relict plants are expected to have their seeds germinate successfully regardless of whether their seeds germinate in spring or have no dormancy. In western China, however, seed germination can be largely impacted due to the short of water availability. Furthermore, 75% and 17% of the studied relict genera are deciduous, respectively. In the case of deciduous trees, the Plum rain in central China and Taiwan Island would help them to grow young leaves in spring, whereas the delayed arrival of the Plum rain in western China would retard the development of new leaves. Deciduous trees, lacking physiological adaptation to seasonal drought, would be particularly impacted. The lack of such a physiological adaptation is to be expected for genera that grew under climates without seasonal drought for millions of years. Several authors have observed that many relict vascular plant species grow preferentially or exclusively in riparian zones^27^,^28^,^29^. This is in line with our observation that relict plants prefer wet climates and a shorter dry season.

Palaeoclimatic reconstructions demonstrate that the European climate was humid in the Eocene, with drier conditions only in the late Oligocene and post-Pliocene^30^. In Siberia and the Russian Far East, climatic conditions were wet during the Oligocene, Miocene and early Pliocene, with a drying trend occurring during the late Miocene^31^,^32^ or late Pliocene^33^. In East Asia, it was humid especially in its eastern portion during the Paleogene, presumably associated with a monsoon-like climate^34^,^35^,^36^. The aridification of central Asia was thought to have begun around the late Miocene^31^,^32^,^37^. In North America, equable and humid conditions occurred during the Paleogene^38^,^39^, and the drying trend did not take place until the latest Miocene^32^. Climatic models also indicate that the climate in the Northern Hemisphere was wet during the Paleogene^40^,^41^. All these studies jointly suggest that the Northern Hemisphere generally had humid conditions during the Paleogene, and began to become less humid or even dry after the late Miocene to late Pliocene. This appears to be consistent with the widespread presence of Cenozoic relict plants in the Northern Hemisphere during the Paleogene and early Neogene. As the climate became cooler and drier in most regions of the Northern Hemisphere, the distribution of relict plants probably contracted correspondingly. These plants probably evolved in a humid environment and at present survive only in those regions where humid conditions have remained to the present day. The palaeoclimatic data do not contradict our hypothesis that Cenozoic relict genera require a humid climate. Plants not adapted to the increasing seasonal drought might have suffered from the winter drying in China; they either retreated to areas suitable for their survival or went to extinction^42^. Because most regions of China experienced an intensification of the dry season largely associated with the onset of the Asian winter monsoon, there was a contraction of ecologically suitable areas. Relict genera survived principally in central China and Taiwan Island, two areas with much less pronounced winter drought. Admittedly, the drying trend is not the only possible cause of plant retreat or extinction; the cooling trend may also have played a role in leading to the disappearance of relict genera from the regions they once occupied.

Mountainous areas have often been cited as suitable refugia because they provide a wide array of sheltered habitats where conditions are often milder and moister^13^,^43^,^44^,^45^,^46^. The altitudinal gradient in mountainous areas allows plant species to shift their distributions during climatic changes^13^,^44^,^47^,^48^. Cenozoic relict plants also often grow in valley bottoms or deep ravines with various slopes where the instability of the sites limits the competition against other plant species^49^. Central China and Taiwan Island also represent mountainous areas and are characterized by complex topography. Large mountains in these two regions can provide suitable refugia for relict plants. Although there are many other mountainous regions in China, they do not gather so many relict genera. This may be due to the fact that climatic conditions in central China and Taiwan Island are regionally specific, i.e., a moister environment with a shorter dry season as described above.

## Methods

### Modern climate of China

The modern climatic data of China were taken from a high-resolution worldwide gridded dataset^50^. Climatic parameters of importance, including spring precipitation (March, April, and May) and autumn precipitation (September, October, and November), were then computed from monthly values.

### Relict genera and their distributions

In this study, we considered woody Cenozoic relict genera growing in China. Most of them have an excellent Cenozoic fossil record in the Northern Hemisphere, e.g., *Metasequoia*^51^,^52^. The fossil record was checked from the literature^5^,^52^. Herein 65 genera representing both gymnosperms (12 genera) and angiosperms (53 genera) were included (Supplementary Table S1). Some genera whose distributions slightly extend beyond China, such as *Diplopanax* in adjacent Vietnam and *Glyptostrobus* in adjacent Laos and Vietnam, are also included. We avoided using the family level as this category is too broad to allow for inferences about climatic requirements of the fossils based only on their extant relatives. On the other hand, it is often impossible to pin-point which extant species is most closely related to the fossil, which also makes a comparison at the specific level incredible. For this reason, the generic level was chosen, which results in a fairly continuous fossil record and allows the climatic requirements of the fossil taxa to be confidently inferred.

The distribution of the Cenozoic relict genera at the county level was checked using the Flora of China and Chinese Virtual Herbarium. Taiwan Island was considered as only one geographic unit. Here we used the term refugia to designate those areas where relict genera are found. This term is appropriate because the sites where the relict genera are found contrast with the widespread distribution they had in the geological past. The distribution of these relict genera was then plotted on a Chinese map using a geographic information system as implemented in the software ArcGIS 9.3 (Environmental Systems Research Institute, Inc.). The number of relict genera present in each county was then compiled. A hotspot analysis was carried out to find the regions where these relict genera are concentrated.

### Species distribution model

In order to better understand the factors controlling the distribution of relict plants, species distribution models were constructed for each studied genus. In the first analysis, 23 climatic parameters were tested, i.e., Bio1 (annual mean temperature), Bio2 (mean diurnal range), Bio3 (isothermality), Bio4 (temperature seasonality), Bio5 (maximum temperature of warmest month), Bio6 (minimum temperature of coldest month), Bio7 (temperature annual range), Bio8 (mean temperature of wettest quarter), Bio9 (mean temperature of driest quarter), Bio10 (mean temperature of warmest quarter), Bio11 (mean temperature of coldest quarter), Bio12 (annual precipitation), Bio13 (precipitation of wettest month), Bio14 (precipitation of driest month), Bio15 (precipitation seasonality), Bio16 (precipitation of wettest quarter), Bio17 (precipitation of driest quarter), CMMT (cold month mean temperature), WMMT (warm month mean temperature), Pspring (spring precipitation), Psummer (summer precipitation), Pautumn (autumn precipitation) and Pwinter (winter precipitation). All climate grids were used at a resolution of 10 arc-minutes. The parameters Bio1 to Bio17 were taken directly from the world climate website^50^. Other parameters were reconstructed as combinations of monthly parameters available at the world climate website^50^. The second analysis included only those parameters that were predominant in the first analysis: Bio4, Bio7, CMMT, Pspring, Psummer, Pautumn and Pwinter. Other parameters that are too well correlated with these ones were not included, e.g., Bio1 and CMMT that show very strong correlation. Both analyses were performed using Maxent^53^,^54^.

## Additional Information

**How to cite this article**: Huang, Y. *et al*. Distribution of Cenozoic plant relicts in China explained by drought in dry season. *Sci. Rep*. **5**, 14212; doi: 10.1038/srep14212 (2015).

## Supplementary Material

Supplementary Information

## Figures and Tables

**Figure 1:**
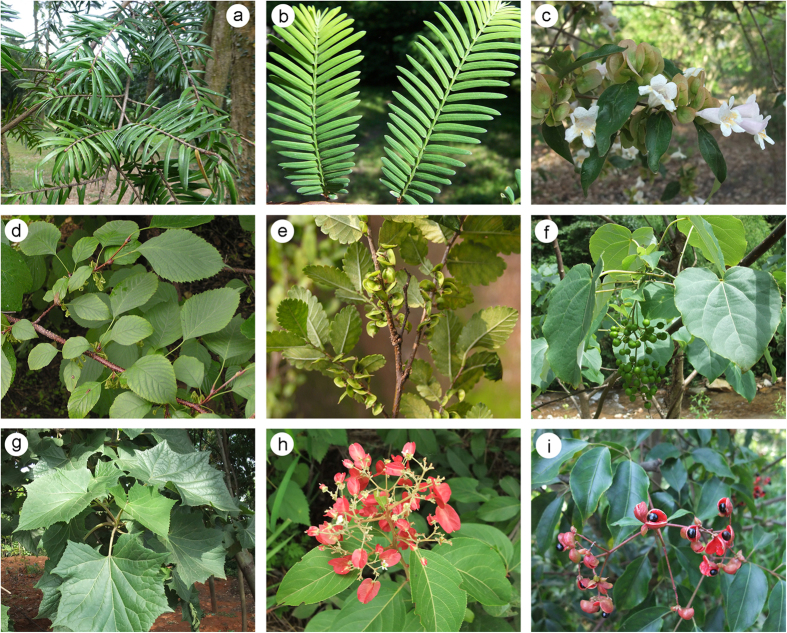
Selected representatives of Cenozoic relict genera in China: (**a**) *Keteleeria*, (**b**) *Metasequoia*, (**c**) *Dipelta*, (**d**) *Euptelea*, (**e**) *Hemiptelea*, (**f**) *Idesia*, (**g**) *Toricellia*, (**h**) *Tripterygium*, (**i**) *Euscaphis*. Image (*b*) was provided by Dr. Li Wang from Xishuangbanna Tropical Botanical Garden, Chinese Academy of Sciences, and images (**c**–**i**) were provided by Dr. Zhuo Zhou from Kunming Institute of Botany, Chinese Academy of Sciences.
