# Supplementary material for: Distribution of Cenozoic plant relicts in China explained by drought in dry season
Source: Sci Rep. 2015 Sep 15;5:14212. doi: 10.1038/srep14212 (PMC4572930; doi:10.1038/srep14212)
Supplement: Supplementary Information [file srep14212-s1.pdf]

Supplementary Information

Manuscript title: Distribution of Cenozoic plant relicts in China explained by drought in dry season

Author list: Yongjiang Huang, Frédéric M.B. Jacques, Tao Su, David K. Ferguson, Hui Tang, Wenyun Chen & Zhekun Zhou*

Supplementary Table S1. The Ceonozic relict genera considered in this study.

Genus	Family	Phenology	Time of seed germination*
Amentotaxus	Taxaceae	Evergreen	April
Amesiodendron	Sapindaceae	Evergreen	March to April
Apterosperma	Theaceae	Evergreen	Unknown
Archiboehmeria	Urticaceae	Evergreen	Unknown
Bretschneidera	Akeniaceae	Deciduous	Spring
Camptotheca	Nyssaceae	Deciduous	March to April
Cathaya	Pinaceae	Evergreen	April to June
Cephalotaxus	Taxaceae	Evergreen	April
Cercidiphyllum	Cercidiphyllaceae	Deciduous	Seed not dormant
Choerospondias	Anacardiaceae	Deciduous	January to March
Corylopsis	Hamamelidaceae	Deciduous	December to January
Craigia	Malvaceae	Deciduous	Unknown
Cryptomeria	Cupressaceae	Evergreen	Unknown
Cunninghamia	Cupressaceae	Evergreen	Unknown
Cyclocarya	Juglandaceae	Deciduous	Spring
Davidia	Nyssaceae	Deciduous	March to April
Decaisnea	Lardizabalaceae	Deciduous	Unknown
Dipelta	Caprifoliaceae	Deciduous	Unknown
Diplopanax	Nyssaceae	Evergreen	Unknown
Dipteronia	Aceraceaee	Deciduous	April
Disanthus	Hamamelidaceae	Deciduous	Unknown
Eucommia	Eucommiaceae	Deciduous	Winter and spring
Euptelea	Eupteleaceae	Deciduous	April to May
Eurycorymbus	Sapindaceae	Deciduous	Seed not dormant
Euryodendron	Pentaphylacaceae	Evergreen	Unknown
Euscaphis	Staphyleaceae	Deciduous	March to April
Fortunearia	Hamamelidaceae	Deciduous	April
Glyptostrobus	Cupressaceae	Semi-evergreen	Unknown
Hemiptelea	Ulmaceae	Deciduous	May
Heptacodium	Caprifoliaceae	Deciduous	Unknown
Hovenia	Rhamnaceae	Deciduous	March to April
Idesia	Flacourtiaceae	Deciduous	Unknown
Keteleeria	Pinaceae	Evergreen	Seed not dormant
Koelreuteria	Sapindaceae	Deciduous	Unknown
Kolkwitzia	Caprifoliaceae	Deciduous	Unknown
Malania	Ximeniaceae	Evergreen	Unknown
Melliodendron	Styracaceae	Deciduous	Unknown
Metasequoia	Taxodiaceae	Deciduous	Seed not dormant
Monimopetalum	Celastraceae	Deciduous	Spring
Ostryopsis	Betulaceae	Deciduous	Unknown
Parapyrenaria	Theaceae	Evergreen	Unknown
Paulownia	Paulowniaceae	Deciduous	March to April

Phellodendron	Rutaceae	Deciduous	Unknown
Platycarya	Juglandaceae	Deciduous	April to May
Poliothyrsis	Salicaceae	Deciduous	Unknown
Poncirus	Rutaceae	Semi-evergreen	April to May
Pseudolarix	Pinaceae	Deciduous	March to April
Pseudotaxus	Taxaceae	Evergreen	Unknown
Pteroceltis	Ulmaceae	Deciduous	Spring
Pterostyrax	Styracaceae	Deciduous	Unknown
Schizophragma	Hydrangeaceae	Deciduous	Unknown
Semiliquidambar	Altingiaceae	Deciduous or evergreen	Unknown
Sinofranchetia	Lardizabalaceae	Deciduous	Unknown
Sinojackia	Styracaceae	Deciduous	Spring
Sinopanax	Araliaceae	Evergreen	Unknown
Sinowilsonia	Hamamelidaceae	Deciduous	Unknown
Taiwania	Cupressaceae	Evergreen	Unknown
Tapiscia	Tapisciaceae	Deciduous	March to April
Tetracentron	Tetracentron	Evergreen	May
Tetrapanax	Araliaceae	Evergreen	March to August
Tetrathyrium	Hamamelidaceae	Evergreen	Unknown
Thujopsis	Cupressaceae	Evergreen	March to May
Toricellia	Toricelliaceae	Deciduous	Unknown
Tripterygium	Celastraceae	Deciduous	Unknown
Weigela	Caprifoliaceae	Deciduous	Unknown

*Information of seed germination is based on "Seeds of Woody Plants in China"[1].

Related reference

1. National Service Center for State-Owned Forest Farms and Forestry Seed Seedling Affairs of the Forestry Administration. Seeds of woody plants in China. (Chinese Forestry Publishing House, Beijing, 2003).

Supplementary Table S2. Percentage contribution of each climatic parameter to explain the geographic distribution.

Genus	Bio4 (℃)	Bio7 (℃)	CMMT (℃)	Pautumn (mm)	Pspring (mm)	Psummer (mm)	Pwinter (mm)
Amentotaxus	17.8	51	17.3	1.4	10.5	1.8	0.1
Amesiodendron	1.8	33.8	40.8	0.4	14.8	6.5	1.9
Apterosperma	2.8	3.4	25.8	1.1	62.8	3.1	1
Archiboehmeria	2.8	17	47.8	15.1	14.1	1	2.2
Bretschneidera	17.4	1.6	0.7	0.7	79.1	0.3	0.3
Camptotheca	10.7	6.7	4.1	23.2	33.5	19.2	2.6
Cathaya	28.3	6.2	2.3	7	38.8	1	16.3
Cephalotaxus	1.6	4	14.7	77.9	0.6	0.6	0.6
Cercidiphyllum	7.8	6.4	11.2	40.1	18.4	2.2	13.8
Choerospondias	2.5	53.9	32.4	1.8	2	0.7	6.8
Corylopsis	12.9	34.5	3.6	44.2	0.9	3.9	0.1
Craigia	3.8	34.2	17.1	2	26.7	3.9	12.3
Cryptomeria	1.3	23.7	2.3	63.5	1.7	1.1	6.4
Cunninghamia	3.4	33.1	2	53	4.3	1	3.2
Cyclocarya	3.1	10.2	3.2	3.5	70.5	9	0.6
Davidia	15.4	17.2	13.7	30.5	11.4	0.6	11.2
Decaisnea	16.3	29.1	5.6	34.7	0.8	1.4	12.1
Dipelta	12.1	28.6	13.1	24.7	1.8	0.3	19.4
Diplopanax	12.4	28.9	2.6	4.6	41	5	5.6
Dipteronia	5.3	7.3	16.2	59.6	0.7	0.7	10.1

Disanthus	11.8	21.1	8	7.2	21.5	0.9	29.5
Eucommia	6.8	12.2	4.1	47.7	19.3	3.8	6.1
Euptelea	2.5	4.5	11.8	24.3	42.7	0.5	13.8
Eurycorymbus	14.3	17.2	1.9	1.2	54	0.3	11.1
Euryodendron	1	2.4	21.9	1.3	66.9	6.2	0.2
Euscaphis	2.7	7.7	21.1	65.3	2.2	0.9	0.2
Fortunearia	14.7	1.4	30.2	23.6	28.5	0.9	0.8
Glyptostrobus	15.9	23.6	1.7	6	31.5	9.3	11.9
Hemiptelea	17.5	9.4	26.3	3.3	2.6	25.7	15.2
Heptacodium	10.5	1.6	3.2	51.8	6	6.4	20.5
Hovenia	3	6.8	63.9	15.5	1.9	8.9	0.1
Idesia	1.7	21.1	2.3	13.2	51.2	10.2	0.4
Keteleeria	4.3	29.8	1.2	57.8	2.9	3.3	0.8
Koelreuteria	1.3	3.1	31.7	22.7	11.6	26.7	3
Kolkwitzia	22.2	1.7	38.3	30.4	0.7	3.4	3.3
Malania	11.1	36.3	17.3	7	28.1	0.3	0
Melliodendron	15.3	18.3	13.2	4.8	30.4	0.5	17.6
Metasequoia	7.8	0.1	2.5	35.7	45.4	0.5	7.9
Monimopetalum	11.7	9.8	3	4.5	20.7	2	48.3
Ostryopsis	8.7	12.2	12.9	31	9.8	1.8	23.7
Parapyrenaria	6.5	4.9	80.8	6	0.1	1.2	0.4
Paulownia	5.2	5.1	14	73	0.9	1.5	0.3
Phellodendron	18.1	6.8	4.7	8.7	1.7	57.9	2
Platycarya	6.6	0.6	24.7	60.8	5.5	0.8	1
Poliothyrsis	7	0.8	11.2	25.1	44.9	7.1	4
Poncirus	14.2	0.5	41.9	3.1	27.6	1.1	11.7
Pseudolarix	6.7	1.6	35.5	0.9	36.5	15.3	3.5
Pseudotaxus	19.7	1.4	1.2	0.9	52.7	4.9	19.1
Pteroceltis	12.5	7.3	12.5	20	25	18.8	3.8
Pterostyrax	10	23.8	2	0.3	62.4	0.7	0.8
Schizophragma	5.5	3.4	5.7	39.3	29.1	16.9	0.2
Semiliquidambar	9.4	22.1	22.7	1.1	24.5	7.4	12.9
Sinofranchetia	3.4	11.3	17.1	35.9	21.8	1.9	8.5
Sinojackia	7.3	31.5	1.9	29.5	6.9	10.1	12.9
Sinopanax	14.4	69.1	0.3	0.3	7.6	6.8	1.4
Sinowilsonia	13.6	2.4	49.4	10.4	16.6	0.3	7.3
Taiwania	19.6	32.6	7.6	8.6	16.5	0.1	14.9
Tapiscia	5	23.5	8.8	55.2	4.8	1.2	1.6
Tetracentron	26.5	47.6	9.1	1	1.7	6.6	7.6
Tetrapanax	12.1	28.7	7	34.3	5.1	4.8	8
Tetrathyrium	7.3	6.6	47.4	0.1	0	5.4	33.2
Thujopsis	12.8	13	16.8	4.9	6.6	11.2	34.8
Toricellia	10.7	31.6	10.8	28.4	6.8	1.6	10.1
Trypterygium	8.8	2	1.1	6.8	35.2	43.7	2.4
Weigela	33.9	2.7	1.7	2.2	18.6	40.8	0.1

Supplementary Table S3. Permutation importance of each climatic parameter to explain the geographic distribution.

Genus	Bio4 (℃)	Bio7 (℃)	CMMT (℃)	Pautumn (mm)	Pspring (mm)	Psummer (mm)	Pwinter (mm)
Amentotaxus	38.6	5.4	17.7	6.4	22.2	8.8	0.9
Amesiodendron	2.5	20.7	32.9	0.7	29.7	1.6	11.9
Apterosperma	12.2	23.4	3.5	6.4	50.2	2.9	1.4
Archiboehmeria	3.6	10.9	60.4	8.8	12.4	0.6	3.3
Bretschneidera	15.4	3.7	2.1	1.6	72.7	3.5	1.1

Camptotheca	17.5	3.2	4.5	6.4	12	46	10.4
Cathaya	42.7	6.3	3.3	2.4	14.9	0.8	29.7
Cephalotaxus	9.9	4.8	20.5	35.4	14.6	7.9	6.9
Cercidiphyllum	7.9	2.9	6.7	62	8.4	8.4	3.8
Choerospondias	17.2	24.1	17.2	6.1	22.1	6.1	7.2
Corylopsis	33.6	9.7	13.2	13.8	11	13.8	4.8
Craigia	1.2	18.9	11.1	1.4	40.5	4.5	22.4
Cryptomeria	9.3	37.3	7.5	16.3	5.6	11.7	12.4
Cunninghamia	13.1	21.6	9.8	10.9	22.5	10.6	11.6
Cyclocarya	7.9	25.1	4.8	7.6	35.5	18.3	0.8
Davidia	26	8.4	19.4	5.5	11.4	7.7	21.6
Decaisnea	7.1	8.2	4.8	65.9	0.7	10.1	3.2
Dipelta	2.7	12.6	27.8	11.7	5.8	2.8	12.2
Diplopanax	36.9	3.4	7.9	10.7	24.7	8.2	8.3
Dipteronia	4.6	4.3	12.3	66	1.2	2.3	9.3
Disanthus	15	3.4	18.2	7.4	12.5	6.1	37.4
Eucommia	12.9	13	6.4	40.1	4.9	7.1	15.6
Euptelea	12.1	2.2	18.5	29.7	24.8	5.6	7
Eurycorymbus	27.1	3.8	9.5	3.3	18.2	6.5	31.7
Euryodendron	0.1	26.3	3.9	5.9	57.1	6.6	0.1
Euscaphis	21.1	14.6	26.5	13.4	9.5	11.8	3
Fortunearia	6.7	7.1	13.7	59.1	6.3	1.7	5.4
Glyptostrobus	10.6	30.1	2.3	15.5	18	6	17.4
Hemiptelea	19.9	1.5	29.6	2.5	8.5	11.7	26.3
Heptacodium	3.1	1.6	8.3	44.3	3.8	0.3	38.6
Hovenia	9.4	13.6	34.7	1.5	24.5	10.2	6.2
Idesia	19.1	5.3	8.6	5.6	37.5	18.1	5.8
Keteleeria	10.9	15	6.6	41.2	6.3	14.2	5.8
Koelreuteria	5.7	7.1	21.1	12.3	26.3	14.1	13.5
Kolkwitzia	37.8	16.7	15.3	8.6	6.8	1.3	13.5
Malania	8.5	3	12.1	10.3	65.3	0.7	0
Melliodendron	22.4	23.2	2	5.6	13	1.3	32.6
Metasequoia	1.8	2.8	0.3	28.9	57.1	0.6	8.6
Monimopetalum	0.7	1.6	6	3.5	87	0.3	0.7
Ostryopsis	12.9	10.1	18.5	18.4	12.6	4.3	23.2
Parapyrenaria	0	54.4	43.8	0.3	0.4	0.2	0.9
Paulownia	14	7.4	19.4	32.3	10.2	9.3	7.4
Phellodendron	27.8	8.5	2.5	7.2	3.2	49.3	1.5
Platycarya	17.1	3.4	30.8	14.7	15.1	11.5	7.6
Poliothyrsis	10	6.1	11.9	59.2	4.7	5.3	2.9
Poncirus	14.7	1	17.3	11.8	5.2	4.8	45.1
Pseudolarix	9.3	1.8	8.2	5.7	15.3	42.7	17
Pseudotaxus	36.8	0.8	2.1	4.3	5.6	30.3	20.2
Pteroceltis	21.6	6.5	11.2	13.2	5.4	33.1	8.9
Pterostyrax	20.3	8.6	8.3	4.7	48.6	0.5	9
Schizophragma	24.4	12.1	9.1	3.5	22.8	27.1	1.1
Semiliquidambar	29	12.2	15.7	3.7	26.7	5.2	7.6
Sinofranchetia	1.8	5.5	4.2	69.2	4.5	11.3	3.5
Sinojackia	11.3	22.3	3.8	4.3	47.1	1.9	9.4
Sinopanax	0	98.6	0.3	0.2	0.1	0	0.6
Sinowilsonia	50.7	15.1	21.9	4.6	1.4	0.2	6.1
Taiwania	50.3	6.5	7.7	0.5	3.1	1.3	30.5
Tapiscia	25.9	10.1	17.2	6.3	24.5	2.5	13.5
Tetracentron	26	23.4	14.5	0.9	10.5	14.5	10.1
Tetrapanax	19.1	2.2	12.8	5.6	19.8	24.6	16
Tetrathyrium	1.9	0	92	0	0	0.2	5.9
Thujopsis	19.9	22.1	27	4.7	2.5	7.7	16.2

Toricellia	19.3	9.9	13.9	15.6	13.2	16.5	11.6
Trypterygium	20.3	7.4	8.7	13.2	36.1	11.9	2.5
Weigela	30	3.2	8.1	5.6	28.2	24.6	0.3

Supplementary Table S4. Number of times each parameter best explains the geographic distribution of relict genera in China.

Parameter	Percent contribution	Permutation importance
Bio4	0	14
Bio7	10	5
CMMT	11	8
Pautumn	19	12
Pspring	19	15
Psummer	3	6
Pwinter	3	5
